# Airborne Bacterial Community Composition According to Their Origin in Tenerife, Canary Islands

**DOI:** 10.3389/fmicb.2021.732961

**Published:** 2021-10-14

**Authors:** Cristina González-Martín, Carlos J. Pérez-González, Elena González-Toril, Francisco J. Expósito, Ángeles Aguilera, Juan P. Díaz

**Affiliations:** ^1^Instituto Universitario de Enfermedades Tropicales y Salud Pública de Canarias, Universidad de La Laguna, San Cristóbal de La Laguna, Spain; ^2^Departamento de Matemáticas, Estadística e Investigación Operativa, Universidad de La Laguna, San Cristóbal de La Laguna, Spain; ^3^Centro de Astrobiología (INTA-CSIC), Instituto Nacional de Técnica Aeroespacial, Torrejón de Ardoz, Spain; ^4^Departamento de Física, Universidad de La Laguna, San Cristóbal de La Laguna, Spain

**Keywords:** airborne, bacteria, 16S rRNA, Tenerife (Canary Islands), wind back trajectories, seasons, next-generation sequencing – NGS

## Abstract

Microorganisms are ubiquitous in the environment, and the atmosphere is no exception. However, airborne bacterial communities are some of the least studied. Increasing our knowledge about these communities and how environmental factors shape them is key to understanding disease outbreaks and transmission routes. We describe airborne bacterial communities at two different sites in Tenerife, La Laguna (urban, 600 m.a.s.l.) and Izaña (high mountain, 2,400 m.a.s.l.), and how they change throughout the year. Illumina MiSeq sequencing was used to target 16S rRNA genes in 293 samples. Results indicated a predominance of Proteobacteria at both sites (>65%), followed by Bacteroidetes, Actinobacteria, and Firmicutes. Gammaproteobacteria were the most frequent within the Proteobacteria phylum during spring and winter, while Alphaproteobacteria dominated in the fall and summer. Within the 519 genera identified, *Cellvibrio* was the most frequent during spring (35.75%) and winter (30.73%); *Limnobacter* (24.49%) and *Blastomonas* (19.88%) dominated in the summer; and *Sediminibacterium* represented 10.26 and 12.41% of fall and winter samples, respectively. *Sphingomonas* was also identified in 17.15% of the fall samples. These five genera were more abundant at the high mountain site, while other common airborne bacteria were more frequent at the urban site (*Kocuria*, *Delftia*, *Mesorhizobium*, and *Methylobacterium*). Diversity values showed different patterns for both sites, with higher values during the cooler seasons in Izaña, whereas the opposite was observed in La Laguna. Regarding wind back trajectories, Tropical air masses were significantly different from African ones at both sites, showing the highest diversity and characterized by genera regularly associated with humans (*Pseudomonas*, *Sphingomonas*, and *Cloacibacterium*), as well as others related to extreme conditions (*Alicyclobacillus*) or typically associated with animals (Lachnospiraceae). Marine and African air masses were consistent and very similar in their microbial composition. By contrast, European trajectories were dominated by *Cellvibrio*, *Pseudomonas*, *Pseudoxanthomonas*, and *Sediminibacterium*. These data contribute to our current state of knowledge in the field of atmospheric microbiology. However, future studies are needed to increase our understanding of the influence of different environmental factors on atmospheric microbial dispersion and the potential impact of airborne microorganisms on ecosystems and public health.

## Introduction

The atmosphere is a dynamic and diverse environment that contains abiotic (chemical elements, compounds of anthropogenic origin, and sand, etc.) and biological components (microorganisms, pollens, seeds, microscopic animals, and algae, etc.) (Westrich et al., 2016). This environment is dynamic, because its composition can change rapidly due to many different factors: wind, storms, rain, geographic features, and location, etc. Air masses move all over the planet transporting all these components with some depositions occurring only a couple meters away and others thousands of kilometers from their sources (Wang et al., 2017). Desert dust that originates in the arid and desert environments of our planet are one of the main sources of aerosolized particles. The Sahara–Sahel region itself accounts for about 50% of that dust, contributing 11–15 Tg of dust per year (Kok et al., 2021).

Up to approximately 25% of aerosolized matter in the atmosphere is biological elements (Jaenicke, 2005). Therefore, the atmosphere is a variable and diverse microbial community (Lighthart, 2000). The number of microorganisms that may be transported over long distances is also diverse and can range from 10^4^ to 10^8^ cells per m^3^ (Bowers et al., 2011). Among them, both animal and plant pathogens may be present, making the atmosphere a potential source of disease (Griffin, 2007; Gonzalez-Martin et al., 2014; Schmale and Ross, 2015).

The presence of microorganisms in the atmosphere not only has consequences for environmental health issues (Karanasiou et al., 2012; Zhang et al., 2016; Tobías and Stafoggia, 2020) but may also affect the climate (Bowers et al., 2009). For instance, their role in the formation of ice nuclei has been considered important at low latitudes, where tropospheric temperatures can be too high to allow abiotic particles to efficiently act as ice nucleators (Spracklen and Heald, 2014), whereas some of the common genera found in airborne samples, such as *Pseudomonas* or *Bacillus*, are known as capable cloud condensation nuclei (Ariya and Amyot, 2004).

The Canary Islands are located between 100 and 500 km off the west coast of Africa. Climate in the archipelago is dominated by the Azores High, characterized by the predominance of trade winds blowing north–northeast, and by the Cold Current of the Canary Islands, which follows a northeast–southwest direction. They are both responsible for the milder-than-normal weather expected according to the islands’ latitude. However, the islands are frequently affected by Saharan dust storms. In 2019, 41% of days were affected by African dust (Pérez et al., 2020), which, depending on its intensity and duration, can have major environmental effects (agronomy, transport, and human health, etc.) (Dorta et al., 2005).

In this study, we determined the airborne bacterial community structure at two different locations, one in an urban environment and the other above high mountain, in Tenerife (Canary Islands), using a next-generation sequencing approach (Behzad et al., 2015; Smets et al., 2016). We aim to understand how airborne bacterial composition may be influenced by different factors including the origin of air masses.

## Materials and Methods

### Samples and Locations

**FIGURE 1:**
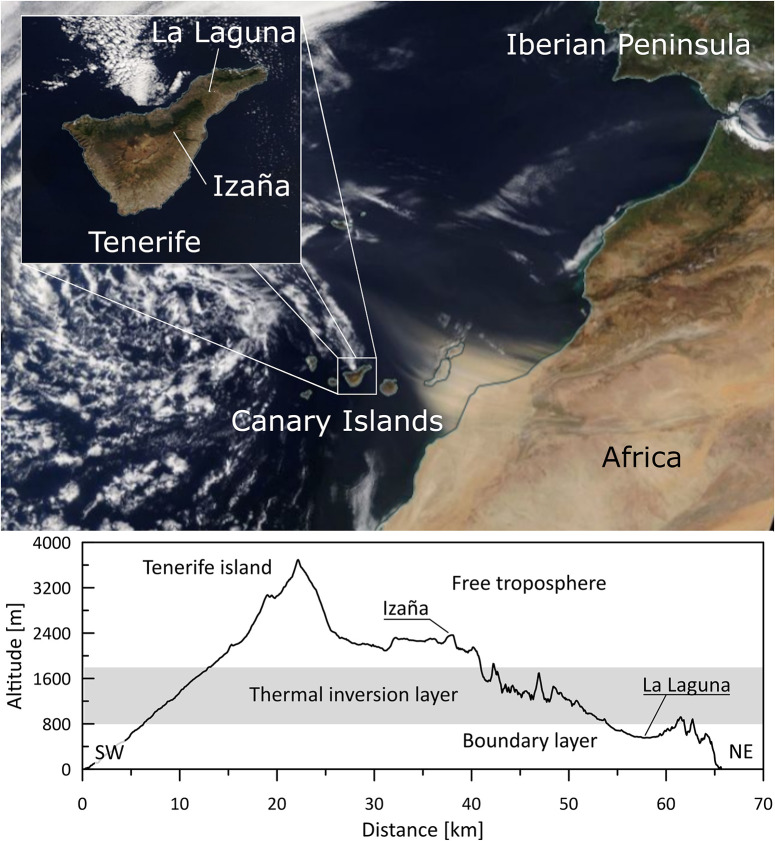
Location of the Izaña and La Laguna stations in Tenerife, Spain (top), and vertical cross section of the island showing the atmospheric vertical layers (bottom).

Sampling was performed in two locations: La Laguna (28° 28′ 44.028″ N; 16° 19′ 17.015″ W) and Izaña (28° 18′ 0.872″ N; 16° 30′ 43.397″ W) in Tenerife, Canary Islands (Figure 1). It was conducted at the same time, at three times a week for a year (10:00 a.m. Mondays, Wednesdays, and Fridays, from June 2017 to June 2018). A total of 293 samples were collected: 144 in La Laguna and 149 samples in Izaña. La Laguna is an urban area located at ∼600 m.a.s.l. in a valley between two mountain ridges. Due to the quasi-permanent presence of the Azores High, this town (150,000 inhabitants) is frequently under the trade wind regime. Temperatures range between 13 and 21°C, and the relative humidity is about 70–80%^1^. The Izaña station is a high mountain site located at ∼2,400 m.a.s.l. on a mountain ridge in roughly the center of the island. Due to its situation, it is mainly affected by the dry descent stream of a Hadley cell, with a relative humidity of approximately 30%–60% and temperatures between 4% and 18°C. Between these stations is a subsidence thermal inversion layer of the subtropical atmosphere of the northeast Atlantic (Font, 1956). This radically separates the atmospheric conditions of both stations and allows us to study the bacterial communities separately, one in the boundary layer and the other in the free troposphere.

Samples were collected at both stations using a Multi-Stage Liquid Impinger (MSLI, Burkard, Rickmansworth, United Kingdom) using 8 ml of sterile 1 × phosphate-buffered saline (PBS) in each chamber (total volume: 24 ml). Sampling was performed during 1 h at a 12 L/min flow rate (total of 720 L of air sampled). Sampling time was previously optimized to avoid excessive evaporation and subsequent sample loss. Remaining 1 × PBS was recuperated in a sterile 50-ml tube and stored in a −80°C freezer until further processing. Recovered volumes ranged from 4 to 24 ml. Control samples were included in the study from the 1 × PBS bottles used to refill the chambers of the sampler. Blank samples were also collected. Samplers were filled with sterile 1 × PBS and incubated for 1 h at the sampling point without pumping. Both control and blank samples were analyzed exactly the same. The MSLI was sterilized between samples using a 10% sodium hypochlorite solution. The solution was sprayed over the apparatus and left for 20 min. Afterward, it was washed with a 1 M sodium thiosulfate solution and distilled water. Subsequently, the compartments were put under ultraviolet light for at least 20 min inside a Class II biological safety cabinet, where they were stored until the next sampling.

### DNA Extraction

Samples were filtered using 0.2-μm Supor PES membrane disc filters (PALL Life Sciences, Port Washington, NY, United States) using an EZ-Fit 3-place manifold (Merck Millipore, Billerica, MA, United States). After filters were allowed to dry, they were cut with sterile scissors over a sterile 55-mm Petri dish. All the pieces were transferred using sterile tweezers to a PowerBead tube from the DNeasy PowerSoil Kit (Qiagen, Germantown, MD, United States). All these steps were carried out inside a Class II biological safety cabinet. DNA extraction was performed following the manufacturer’s instructions, except for some previously published modifications (Jiang et al., 2015); and samples were stored in the -80°C freezer until being shipped to the sequencing service.

### Next Generation Sequencing

Sequencing was performed in the Plataforma de Genómica - Fundación Parque Científico de Madrid. Purified DNA was quantified by PicoGreen, and concentrations ranged from 15 to 100 pg. The first PCR used an input of DNA that ranged from 0.6 to 5 μl, and between 26 and 32 cycles were performed with Q5^®^ Hot Start High-Fidelity DNA Polymerase (New England Biolabs, Ipswich, MA, United States) in the presence of 100 nM of primers for 16S rRNA amplification (5′-ACACTGACGACATGGTTCTACACCTAC GGGNGGCWGCAG-3′ and 5′-TACGGTAGCAGAGACTTGG TCTGACTACHVGGGTATCTAATCC-3′). These primers amplify the V3–V4 region of the 16S rRNA. After the first PCR, a second PCR of 13 cycles was performed with Q5^®^ Hot Start High-Fidelity DNA Polymerase (New England Biolabs) in the presence of 400 nM of primers (5′-AATGATACGGC GACCACCGAGATCTACACTGACGACATGGTTCTACA-3′ and 5′-CAAGCAGAAGACGGCATACGAGAT-[10 nucleotides barcode]-TACGGTAGCAGAGACTTGGTCT-3′) of the Access Array Barcode Library for Illumina Sequencers (Fluidigm, South San Francisco, CA, United States).

The amplicons obtained were validated and quantified by Bioanalyzer, and an equimolecular pool was purified using AMPure beads (Beckman Coulter, Brea, CA, United States) and titrated by quantitative PCR using the Kapa-SYBR FAST qPCR kit for Light Cycler 480 (Merck, Kenilworth, NJ, United States) and a reference standard for quantification. The pool of amplicons was denatured prior to being seeded in a flowcell at a density of 10 pM, where clusters were formed and sequenced using a MiSeq Reagent Kit v3 (Illumina, San Diego, CA, United States), in a 2 × 300 paired-end sequencing run on a MiSeq sequencer. From the initial 293 samples collected, information was obtained for 220 of them (104 from Izaña and 116 from La Laguna). Samples that initially did not produce any PCR signal were either left out of the study or mixed with other samples from previous/next days that matched the same weather conditions.

### Data Processing

The analysis of raw FASTQ sequencing data was performed in multiple steps using the Mothur pipeline v 1.41.1, adopted from the Schloss SOP (Schloss and Westcott, 2011). Illumina MiSeq paired-end sequences were processed to apply quality trimming and removal of low-quality sequences using the publicly released 132 of SILVA reference composite dataset for bacterial, archaeal, and eukaryotic sequences (Pruesse et al., 2007).

To account for possible contaminants associated with the experimental design, filtered taxonomic tables were generated after removing a subset of sequences identified from the extraction-negative and no-template PCR control samples. The remaining sequences were clustered and classified into operational taxonomic units (OTUs) identified at a cutoff of 97% identity. The classification was performed using the RDP training set, which consists of a taxonomical data collection of 12,681 bacterial and 531 archaeal 16S rRNA gene sequences (Cole et al., 2014). In subsequent analyses, a total of 494,759 representative bacterial sequences were assigned at different taxonomic levels (from phylum to genus).

### Statistical Analysis

All statistical analysis and figures were produced with R software (v.4.1.0.), primarily using the statistical package vegan (Oksanen et al., 2020) and the graph package ggplot22 (Wickham, 2016). After the OTUs were obtained, the alpha diversity of bacterial communities was analyzed using the phyloseq package (McMurdie and Holmes, 2013). The numbers of unique OTUs by sampling locations were visualized in a Venn diagram plot using the package VennDiagram (Hanbo, 2018). Kruskal–Wallis tests were applied to analyze differences between bacterial communities. The comparison of sampling units was performed by applying the analysis of similarities (ANOSIM) as well as the non-metric multidimensional scaling (NMDS) and principal coordinates analysis (PCoA) techniques.

### Collection of Climatic Information

**FIGURE 2:**
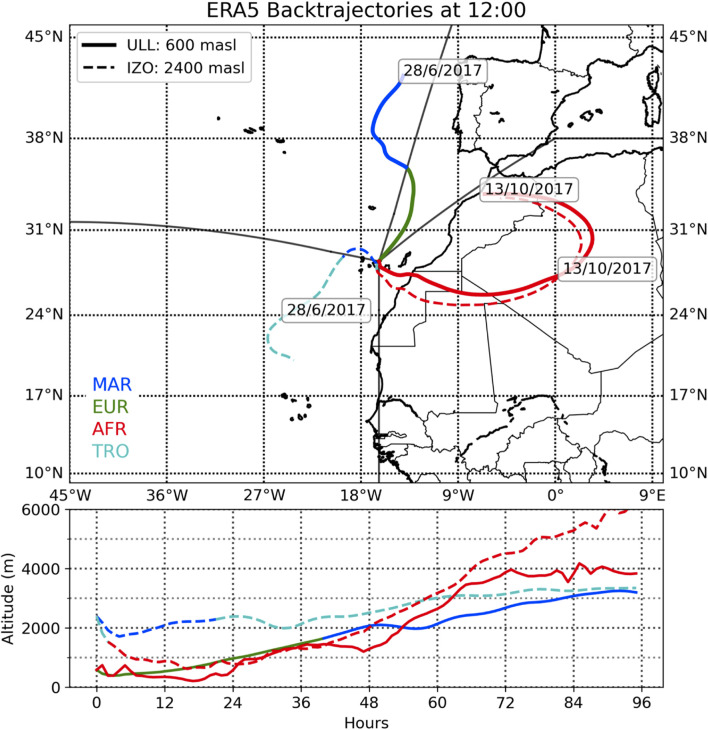
Example of wind back trajectories for the La Laguna (LLA, solid line) and Izaña (IZO, dashed line) stations showing the different behaviors of the air masses that reach the sampling points. The colors of the back trajectories indicate the region through which the air masses flow: Marine (MAR, blue), European (EUR, green), African (AFR, red), and Tropical (TRO, cyan).

Meteorological variables (wind speed, temperature, and rainfall) were obtained from the Agencia Española de Meteorología website (AEMET, 2020). The Azores High dominates the weather in this archipelago bringing air masses from Europe or the Atlantic Ocean depending on the sampling site. In addition, African dust outbreaks or westerly lows frequently occur, with different consequences at both stations, due to air masses arriving from Africa or from the Tropical Atlantic Ocean. Natural episodes of African dust outbreaks were monitored using forecasts provided by the Consejo Superior de Investigaciones Científicas (CSIC) (Pérez et al., 2017, 2018). The origins of the air masses were established and verified by back trajectories (Figure 2) described by the Hysplit-4 model (Stein et al., 2016). This software requires accurate meteorological data as input; and in this case, the fifth-generation atmospheric reanalysis of the global climate data (ERA5), computed by the European Centre for Medium-Range Weather Forecasts (ECMWF) (ERA5, 2017) was used. Following criteria previously described (Díaz et al., 2006) regarding the regions over which the back trajectories of the air masses flow and are loaded with aerosols, the archipelago area was divided into four regions: North Atlantic Ocean, MAR; Europe, EUR; Africa, AFR; and Tropical Atlantic Ocean, TRO. The air masses were also classified into five categories: African (AF), Marine (MA), European (EU), Tropical (TR), and Combined (CB). African corresponded to those aerosols that originated and/or whose trajectories crossed part of the North African continent. Marine air masses had a predominant trajectory over the North Atlantic Ocean, and European ones were considered as those with European and Marine aerosols and whose trajectories crossed the European continent and the Atlantic Ocean before reaching the Canary Islands. Tropical air masses were those with a marine trajectory but originated from the Atlantic Ocean, southwest of the archipelago. The Combined class considered air masses that crossed two to four different regions during their trajectory before reaching the sampling locations. In this work, we have not considered the Combined class, only the four pure types.

## Results

### Overview

Here, we investigated the airborne bacterial communities at two differentiated areas of Tenerife over a year. From the 293 original samples, 220 were finally sequenced: 104 from Izaña and 116 from La Laguna. Samples that did not produce any signal at the first step of the sequencing process were grouped with others of similar characteristics (same back trajectories and meteorological conditions). Control and blank samples did not yield any detectable DNA, and the sequencing process did not produce any data. However, sequences were obtained from no-template PCR control samples, but these were removed from all samples prior to statistical analysis. Total number of reads for all samples reached 29,421,993, an average of 130,186 per sample. After processing, a total of 2,215,797 reads remained, about 9,804 per sample. Clustering at 97% identity yielded 67,185 OTUs (see Supplementary Table 1 for detailed sequencing information).

### Operational Taxonomic Unit Abundance

Sequences were mainly classified as Proteobacteria (88%), followed by Bacteroidetes (3.5%), Actinobacteria (3.4%), and Firmicutes (2.7%). Supplementary Tables 2, 3 provide detailed information about phyla and genera (number of sequences and relative abundances), separately for La Laguna and Izaña, respectively, and only for genera present at both locations.

**FIGURE 3:**
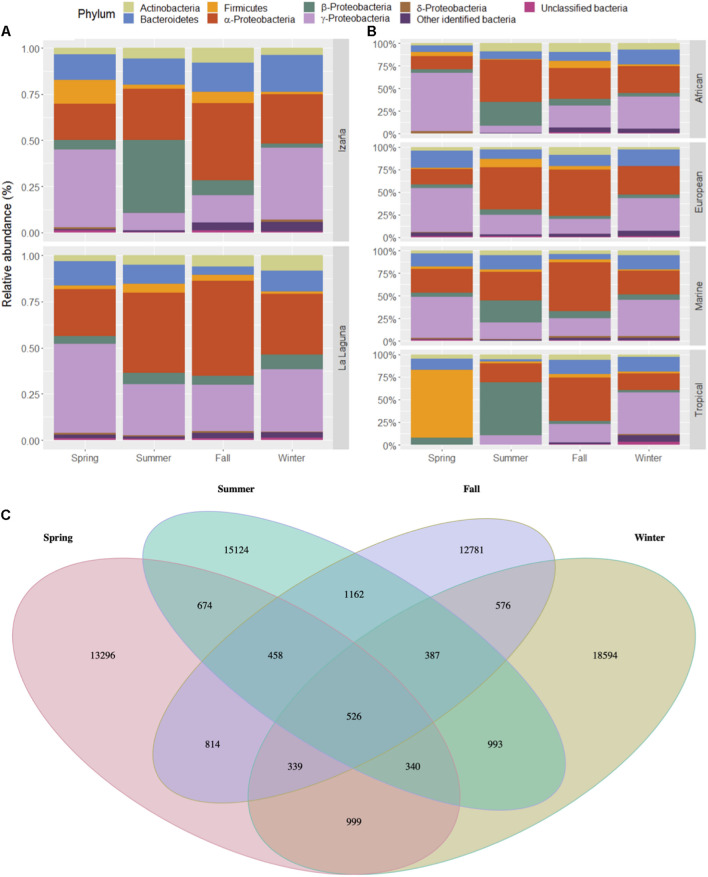
Relative abundances of the most predominant bacterial taxa at the phylum level in both locations by season **(A)** and wind back trajectory **(B)**. Venn diagram of unique and common bacterial phyla by season **(C)**.

Relative abundance of main bacteria phyla, and classes (only for Proteobacteria), are shown in Figure 3A by sampling seasons (see Supplementary Table 4). Between 65 and 75% of air microbiome in the Izaña samples corresponded to Proteobacteria, whereas occurrence of more than 75% of these organisms was observed in the La Laguna samples. Gammaproteobacteria were the most represented class during spring and winter at both locations (33.64–48.50%), while in the fall, it was Alphaproteobacteria (41–51.46%). During the summer, differences were observed between locations, since Alphaproteobacteria were dominant at La Laguna (43.45%), but Betaproteobacteria dominated at Izaña (39.54%). Bacteroidetes phylum presented a relative abundance between 14% and 20% throughout the year in Izaña, while in La Laguna, it was lower than 13%, especially in fall where it ranged from 4 to 5%. Actinobacteria and Firmicutes were the other two main phyla represented, with relative abundances < 10%. Less than 2% of the air microbiome could not be identified as a known taxon. Figure 3B also shows relative abundances, but taking into account the origin of the air masses (see Supplementary Table 5). No major differences were detected, except for the Tropical air masses, which showed a significantly high presence (75%) of the Firmicutes phylum during the spring and the Betaproteobacteria class (58.67%) during the summer. Also, Bacteroidetes showed a significant presence in the fall and winter in Tropical air masses (15.78–16.87%). The other three categories (African, European, and Marine) presented more similar compositions during the spring, fall, and winter, with a predominance of Gammaproteobacteria. Alphaproteobacteria in the summer. However, Betaproteobacteria reached percentages of approximately 20% in African and Marine wind back trajectories for the summer season. In Figure 3C, a Venn diagram is shown, representing the richness of bacterial phyla in the different seasons, with summer and winter presenting the highest number of unique OTUs.

### Bacterial Diversity

**FIGURE 4:**
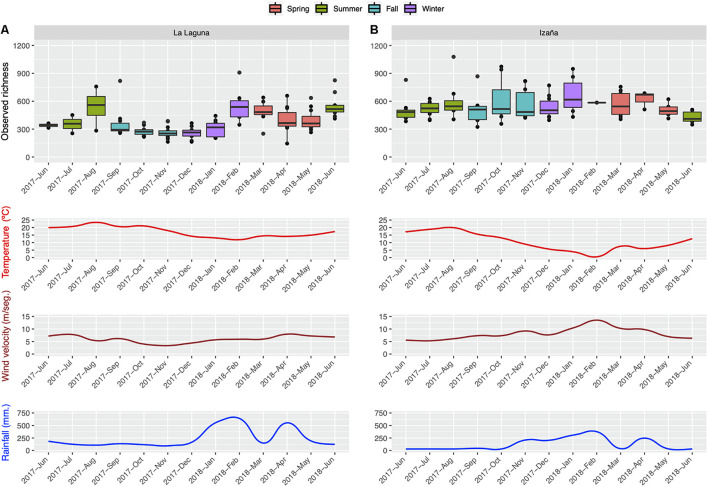
Monthly distribution of observed richness by sampling location, La Laguna **(A)** and Izaña **(B)**, along with monthly average values of temperature and wind speed, and monthly accumulated rainfall.

The monthly distribution of bacterial richness is illustrated in Figure 4. La Laguna (Figure 4A) reached its peak in the summer, while the Izaña (Figure 4B) maximum was in the winter. Spearman’s correlation tests were performed to analyze any links between environmental variables (average temperature and wind speed, and accumulated rainfall) and observed monthly richness, but no significant correlations were detected (data not shown). Regarding temperature, La Laguna showed the highest levels of richness at the highest and lowest temperature values, while in Izaña, richness rose as temperatures decreased. Higher wind velocities also appeared to positively impact prokaryotic richness in Izaña, up to its maximum in January 2018, and heavy rainfall seemed to have a direct effect at both locations.

Alpha diversity estimators, including Chao1 and Shannon indices, were calculated to estimate the average observed richness and biodiversity of the bacterial communities (Supplementary Figure 1). Izaña samples presented a similar diversity in all seasons (Kruskal–Wallis chi-squared = 3.6168, *p*-value = 0.3059), whereas the differences were statistically significant between samples from La Laguna (Kruskal–Wallis chi-squared = 31.82, *p*-value = 5.71e−07). Specifically, Dunn’s test showed that diversity was very similar between the spring and the summer, as well as between fall and winter, but detected significant differences between the warmer (spring + summer) and the cooler seasons (fall + winter) (Supplementary Figure 1A and Supplementary Table 6). Regarding the air mass origin, in samples collected in La Laguna (Supplementary Figure 1B), significant differences (Kruskal–Wallis chi-squared = 15.999, *p*-value = 0.001134) were found between African and Marine wind back trajectories, as well as between the Marine and Tropical air masses (Supplementary Table 7). A significant difference was also detected in the air masses for the samples of Izaña (Kruskal–Wallis chi-squared = 8.8896, df = 3, *p*-value = 0.0308), specifically between the African and Tropical wind back trajectories (Supplementary Table 8).

### Seasonality and Wind Back Trajectory Relationship With Taxonomic Assignment

Over 500 different genera were identified, with five being the most abundant during different seasons of the year (relative abundance > 10%). *Cellvibrio* was the most frequent genera during winter and spring, at 30.73 and 34.75%, respectively. *Limnobacter* (29.49%) and *Blastomonas* (19.88%) prevailed over the other genera detected during the summer. Fall samples produced the highest levels for *Sphingomonas* (17.15%), a genus barely detectable during the other seasons. Fall also had 10.26% relative abundance of *Sediminibacterium* genus, which was also detected in the winter samples with a similar percentage (12.41%). In addition, potential pathogenic bacterial genera were detected. Among the groups with relative abundances over 10%, some genera with known human pathogenic species were *Acinetobacter*, *Enterococcus*, and *Pseudomonas*.

**FIGURE 5:**
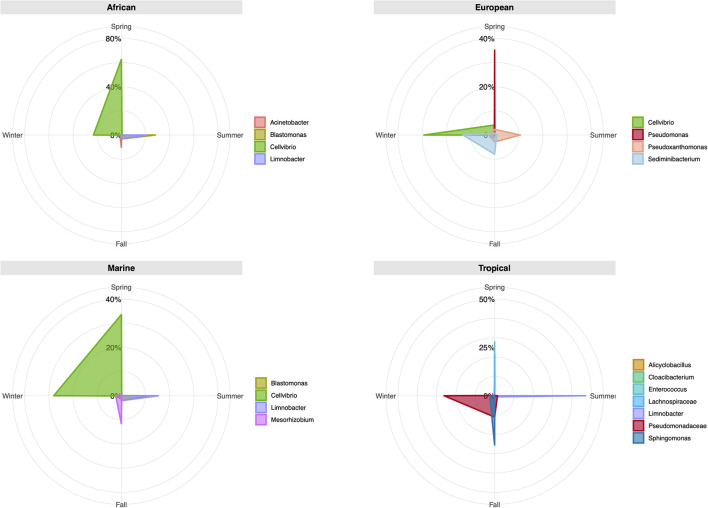
Radar chart presentation of relative operational taxonomic unit (OTU) abundance of taxa at the genus level for different seasons and wind back trajectories. Each chart shows the season levels for the most abundant bacterial genera (>10%). The values along each axis of the radar chart are connected linearly to visualize genera relative abundance as a polygon.

Considering the origin of the air masses, African, and Marine plumes presented a similar composition regarding the most common genera (Figure 5 and Supplementary Table 9). During the spring (30–56%) and winter (22–28%), *Cellvibrio* was the most frequently identified, while in summer, *Blastomonas* and *Limnobacter* dominated the bacterial communities. European air masses, which also showed a high number of *Cellvibrio* OTUs, produced sequence prevalence of over 10% for the genera *Pseudomonas* (35.06%), *Sediminibacterium* (13.00%), and *Pseudoxanthomonas* (10.61%). Tropical plumes also produced a greater number of different genera. The most frequent bacterial genera during spring were *Cloacibacterium*, *Alicyclobacillus*, *Enterococcus*, and the Lachnospiraceae family (ranging between 12 and 28%). The summer was dominated by *Limnobacter* (46.94%), while during fall, *Sphingomonas* dominated (25.35%). Unclassified genera from the Pseudomonadaceae family were detected in the fall (10.98%) and in winter (25.60%).

**FIGURE 6:**
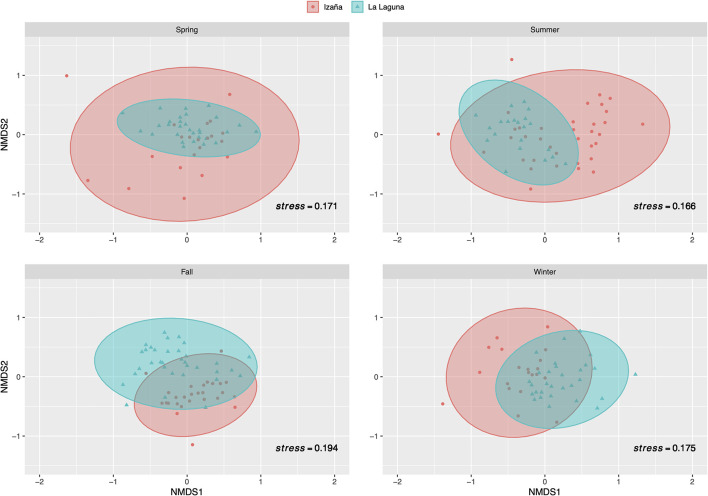
Non-metric multidimensional scaling (NMDS) analysis by season and sampling site. Operational taxonomic units (OTUs) that did not appear more than five times in more than half of the samples were removed. Five most predominant phyla were selected for the analysis. Stress value under 0.2 denotes good ordination of microbial community samples in two dimensions.

Non-metric multidimensional scaling analysis using two dimensions was performed to determine dissimilarity between sampling locations and showed good ordination stress values (a value under 0.2 denotes good ordination of microbial community samples in two dimensions) (Figure 6). La Laguna samples showed a more compact distribution throughout the year, while Izaña samples exhibited a wider pattern, indicating that the prokaryotic community at the high mountain site fluctuated more than the urban location, especially during the spring and summer. During the cooler seasons, both locations presented a more compact allocation, and only in the fall were the Izaña samples surpassed by those collected at La Laguna. ANOSIM and PCoA tests were additionally conducted to determine similarity in the airborne bacterial community between locations and seasons (Supplementary Figures 2, 3, respectively). ANOSIM values ranged between 0.0519 and 0.546, *p*-values < 0.01, except in the spring, when the value was equal to 0.0701, and there was no disaggregation by location. In the other three seasons, especially the fall, community composition tended to cluster by sampling location. The PCoA results based on the Bray–Curtis dissimilarity matrix showed the separation among samples by season for a high percentage of variability within the bacterial community structure. Between 55 and 60% of the variance was explained by the two axes’ components for both locations, La Laguna, and Izaña.

## Discussion

The focus of this research is to gain a deeper understanding of the communities of airborne bacteria in different locations in Tenerife and to determine if there are any links between airborne bacterial community composition and the origin of the air masses. Considering sampling location, we found the high mountain site to be more diverse than the urban one. Significant differences have been previously reported for studies conducted in France (Samaké et al., 2021), Italy (Gandolfi et al., 2015), and China (Xie et al., 2018). In those cases, land use, climate, or diverse urban characteristics influenced the airborne bacterial composition. Our results are more in line with those found by Tanaka et al. (2019) they also collected samples at two sites with different altitudes (23 and 2,839 m.a.s.l.), and they reported a higher fluctuation in the bacterial community composition at the high mountain site (Tanaka et al., 2019).

### Seasonal Variations

Although composition changed throughout the year, it was dominated by bacteria belonging to Proteobacteria. Most of the relevant literature has reported similar findings. Proteobacteria is regularly the most frequent phylum in airborne samples (Cáliz et al., 2018; Aalismail et al., 2020). However, percentages obtained here are significantly higher (>65%) than those of most previous studies (25–51%) (Tringe et al., 2008; Barberan et al., 2014; Prussin et al., 2016; Abd Aziz et al., 2018; Tanaka et al., 2019; Uetake et al., 2019). There are also studies that have reported different phylum abundances; e.g., samples collected in Korea showed Proteobacteria were predominant during non-dust days, while Bacteroidetes were the most frequent during Asian dust days (Cao et al., 2014; Abd Aziz et al., 2018; Núñez et al., 2019).

Different classes within the Proteobacteria phylum predominate over the seasons, while Alphaproteobacteria have been found to prevail in previous reports (Gao et al., 2017; Cáliz et al., 2018; Samaké et al., 2021). Particularly noteworthy is that Gammaproteobacteria were frequently found during the spring and winter (>30%) in this study, in contrast to previously published reports where this group rarely surpassed 15% prevalence (Gao et al., 2017; Cáliz et al., 2018; Yan et al., 2018; Tanaka et al., 2019; Samaké et al., 2021). In fact, in a 7-year study performed in the Pyrenees, relative abundances by main phyla and Proteobacteria classes had similar results to our 1-year analysis, with the exception of Gammaproteobacteria, which only represented 6% compared with the maximum 48.5% in this work (Cáliz et al., 2018). Bacteroidetes, Firmicutes, and Actinobacteria abundances were more variable throughout the year and did not show a clear trend. Other frequent phyla previously described in air samples, such as Acidobacteria, Cyanobacteria, Chloroflexi, or Deinococcus-Thermus (Kellogg et al., 2004; Gao et al., 2017; Aalismail et al., 2020), were identified in less than 1% of the sequences in this study. Generally, summer samples showed the highest abundance levels at both sampling sites, similar to trends observed in previous reports (Bowers et al., 2012; Bertolini et al., 2013; Be et al., 2015; Genitsaris et al., 2017; Núñez et al., 2021), but the highest number of unique OTUs for a given season corresponded to winter in this study. However, when abundance was classified by season and air mass source, summer showed the highest abundance for the Marine and Tropical trajectories, while for the African and European trajectories, it was winter. This agrees with the current postulation that airborne communities are influenced by multiple factors, both meteorological and environmental (Uetake et al., 2019; Núñez et al., 2021).

### Influence of Wind Back Trajectories

Significant differences were detected between African and Tropical air masses for both sampling sites. Previous authors have described how diversity may increase under the influence of desert dust (Tang et al., 2016), while others have reported how the Shannon diversity index may decrease after a dust event (Guo et al., 2018). In this study, such differences were mostly due to the diversity described for Tropical air masses, not African ones. Differences between African and Marine air masses in La Laguna may have been influenced by the number of samples within each category, since Marine air masses in the urban site almost quadrupled the African ones. Moreover, there were some rare genera only present in the Marine samples (e.g., *Anaerococcus*, *Opitutus*, *Niabella*, *Rothia*, *Ezakiella*, *Veillonella*, *Sutterella*, and *Sphingorhabdus*). Some have been previously described in air samples linked to an oceanic origin, for example, *Opitutus* (Uetake et al., 2019), while others were related to soil environments, like *Sutterella* (Gusareva et al., 2019). However, for the whole set of samples, Marine and African air masses bacterial composition was very similar, as illustrated in the radar plot in Figure 5. We consider that, given the insularity of the archipelago and its proximity to the African continent, there is a high interconnection between these two types of air masses. Future studies should include soil analyses from source regions, which would allow better comparisons and discrimination regarding the origin of certain microbes.

### Environmental Impact on Airborne Bacterial Richness and Diversity

Observed richness showed quite a different trend at both sampling sites. In La Laguna, the lowest diversity levels were during the fall and winter, increasing again from late in the winter season. To the contrary, in Izaña, bacterial diversity presented its peak during the cold seasons, showing an inverse correlation with temperature. No significant correlation to environmental variables was found in the sample sets analyzed. As some authors have stated before, especially using next-sequencing technologies (Bowers et al., 2011; Shin et al., 2015), these factors vary throughout the year and follow different patterns (Fahlgren et al., 2010; Whon et al., 2012; Gandolfi et al., 2015). However, there are authors that have reported a direct and positive correlation between temperature and bacterial diversity (Zhen et al., 2017; Núñez et al., 2021). These studies have been conducted in continental areas with stable climatic conditions throughout the year, unlike Tenerife, in the Canary Islands, which is an archipelago with very variable seasonal conditions and regions of elevated topography (0–3.718 m.a.s.l.). The influence of wind speed on bacterial richness has been described before, with a positive correlation (Gandolfi et al., 2015). In our particular case, the highest wind velocities corresponded to a snowstorm and heavy rainfall event in February 2018 in Izaña, and it was a defining moment for an observed decreased in richness. Another possible explanation for these results has been described by Uetake and others; although alpha diversity usually decreases after precipitation, they suggest that heavy rain events may increase bioaerosols because of the impact of rain drops on different surfaces (Wang et al., 2016; Joung et al., 2017; Uetake et al., 2019). During the winter season 2017–2018, two intense storms affected both sampling sites, storm Ana in December and storm Emma in late February. This could have led to the increased diversity observed (AEMET, 2020), which marks the completely different trends at both sampling sites over the seasons (Supplementary Figure 3). Also, some authors have reported that variations in local bacterial sources may have a higher impact on airborne communities than local meteorological conditions (Bowers et al., 2011).

Diversity values obtained were similar or higher than previously reported (Cáliz et al., 2018; Smith et al., 2018; Els et al., 2020), yet we could not establish a seasonal pattern. Although the summer showed the highest observed diversity, the Shannon index for this season was the lowest, especially within the Izaña sample set, indicating that the bacterial community identified is not evenly distributed.

### Distinguished Taxonomic Data

In the current study, most of the identified phylotypes are derived from soil and marine environments, as expected due to the geographic condition and the frequent dust events over the archipelago. *Cellvibrio* and *Blastomonas* have been previously reported from air samples collected in Singapore (Gusareva et al., 2019). Conversely, *Limnobacter* and *Sediminibacterium*, the other two most frequent genera identified in this study, have only been reported in water or soil studies (Jia et al., 2015). All these four genera were most abundantly identified in the Izaña samples.

Moreover, the effect of anthropogenic activities could be inferred from the results for some genera, such as *Cloacibacterium*, *Enterococcus*, or the Lachnospiraceae family. Lachnospiraceae have been identified in airborne studies, some analyzed airborne bacterial composition in animal facilities (Hong et al., 2012; Nguyen et al., 2019), and others were more similar to this study, which analyzed airborne communities during diverse atmospheric conditions (Seifried et al., 2015; Smith et al., 2018; Guo et al., 2020). However, in this study, these genera were more abundant at the high mountain site than the urban one, which was especially due to one sample collected on March 9, 2018 (IZ103). The high concentration of bacteria within the Firmicutes group on that particular day from a Tropical air mass may indicate that only the most stress-resistant bacteria are able to withstand the stress resulting from the atmospheric transport to Izaña for that sampling period. That sample was also the only one where the genera *Thermicanus* and *Terrisporobacter* were identified, which contrasts with previous airborne descriptions conducted in built-up environments for *Thermicanus* (Dubuis et al., 2017; Hamoda and Mahmoud, 2019). Moreover, the *Terrisporobacter* genus contains species that are known to be human and animal pathogens (Cheng et al., 2016; Fong et al., 2020).

Recurrent genera usually identified in airborne microbial studies, like *Kocuria*, *Delftia*, *Mesorhizobium*, *Methylobacterium*, and *Paracoccus* (Zhai et al., 2018; Chen et al., 2020), were also detected in our samples, though they were more frequent in the La Laguna samples. There was one genus, *Tepidisphaeara*, only identified in La Laguna, specifically on September 8, 2017 (LL028), which is considered an indicator for algal blooms (Shao et al., 2020). Curiously, during the summer of 2017, Tenerife was affected by multiple episodes of algal blooms along its coast. An abundant genus in the Tropical air masses was *Alicyclobacillus*, a bacteria commonly found in soil and extreme environments, which has been described in previous airborne studies (Korves et al., 2012; Miletto and Lindow, 2015).

Among the 519 genera identified, there were also many potentially hazardous bacteria: *Acinetobacter*, *Bacillus*, *Brucella*, *Enterococcus*, *Neisseria*, *Staphylococcus*, *Streptococcus*, and *Pseudomonas* are among those that contain species that are pathogenic to plants and/or animals (Polymenakou et al., 2008; Li et al., 2020; Bayle et al., 2021). Some of these airborne transmissions have been studied (Leski et al., 2011; Fan et al., 2019; Ruiz-Gil et al., 2020). Also, the genus *Brevundimonas* was among the most abundant genera identified. A species of this genus has been considered an emerging potential pathogen for nosocomial infections (Triadó-Margarit et al., 2019), which, given its global presence and ability to survive under extreme conditions (Dartnell et al., 2010), should be a concern for public health authorities. Some samples also produced a high number of OTUs (>10) for some of the pathogenic genera (e.g., *Escherichia*/*Shigella*, *Staphylococcus*, and *Streptococcus*), but no significant trend was observed that illustrated a specific link to season or air mass origin.

### Concluding Remarks

The description of the airborne bacterial community in two different areas has been achieved for the first time in the Canary Islands, showing great diversity, especially at the high mountain site. This report is the starting point for further characterization of airborne microbial communities that we aim to analyze and establish over a longer term together with their links to different environmental variables. Indeed, previous atmospheric microbiology studies have described how local meteorology may have a greater influence on composition than short/long term transport, meaning that origin may not play such an important role (Uetake et al., 2019; Samaké et al., 2021). However, others have reported a strong correlation between airborne bacteria and aerosol source (Cáliz et al., 2018). Some have hypothesized how urban and industrial sites are influenced by non-seasonal variables, such as human activities, while more rural locations are dominated by, for example, bacteria related to vegetation (Xie et al., 2018). A combination of all these variables can lead to a bacterial community structure in the atmosphere that can be predicted. While each study has their own particularities, the sampling period appears to be a decisive factor in order to obtain strong correlation patterns. More long-term studies are needed that will help explain the variations observed in the airborne microbial communities described throughout the world. In addition, they could help identify airborne bacterial community patterns due to different variables. In both cases, we suggest that a global airborne microbiology network be put in place, so that longer-term datasets could be utilized to produce global scale trends. In a global climate change scenario and considering the impact that microorganisms may have on receptor ecosystems (public health and agronomy, etc.), it is imperative to engage in new scientific advances and alliances that allow us to investigate how and for how long some microbes are transported through various atmospheric routes and remain viable.

## Data Availability Statement

The datasets presented in this study can be found in online repositories. The names of the repository/repositories and accession number(s) can be found below: https://www.ebi.ac.uk/ena/, PRJEB46651.

## Author Contributions

JD, FE, and CG-M designed the study. CG-M performed samplings and laboratory analyses. CP-G, EG-T, and ÁA executed the data analysis. All authors contributed to writing and reviewing the manuscript, as well as approving it for publication.

## Conflict of Interest

The authors declare that the research was conducted in the absence of any commercial or financial relationships that could be construed as a potential conflict of interest.

## Publisher’s Note

All claims expressed in this article are solely those of the authors and do not necessarily represent those of their affiliated organizations, or those of the publisher, the editors and the reviewers. Any product that may be evaluated in this article, or claim that may be made by its manufacturer, is not guaranteed or endorsed by the publisher.
