# Supplementary material for: Airborne Bacterial Community Composition According to Their Origin in Tenerife, Canary Islands
Source: Front Microbiol. 2021 Oct 14;12:732961. doi: 10.3389/fmicb.2021.732961 (PMC8563076; doi:10.3389/fmicb.2021.732961)

Supplementary Material

Table S1. Summary of sequencing data for each sample and control (no-template PCR control samples that produced sequences).

Sample	Make contigs	Sequences screening	Remove chimeras	Subtracted sequences per sample	% Eliminated sequences per sample	Final sequences per sample
Control 03a	205439	55713	47606	205439	100%	0
Control 05a	78668	582	568	78668	100%	0
Control 08a	188164	64358	59237	188164	100%	0
Control 11a	108280	29562	29435	108280	100%	0
Control 12a	109085	31726	31703	109085	100%	0
LL1	128867	55419	55011	118356	91.84%	10511
LL3	117235	44274	44027	88508	75.50%	28727
LL4	88913	35499	35311	65062	73.17%	23851
LL8	122049	57786	56898	114370	93.71%	7679
LL9	106557	43779	43294	86104	80.81%	20453
LL10	113771	43397	43048	88856	78.10%	24915
LL21	92016	37257	36538	74102	80.53%	17914
LL25	96580	47455	47082	94193	97.53%	2387
LL28	128489	45468	43479	98120	76.36%	30369
LL29	119371	57471	56444	111690	93.57%	7681
LL30	117367	46426	45162	105524	89.91%	11843
LL31	103136	38664	37884	89399	86.68%	13737
LL32	110549	53211	52682	109979	99.48%	570
LL33	145334	56308	50209	137978	94.94%	7356
LL34	108358	52956	52637	107894	99.57%	464
LL35	117711	55447	54542	116557	99.02%	1154
LL36	125131	57818	57328	124335	99.36%	796
LL37	111936	54420	53695	111463	99.58%	473
LL38	108937	53002	52613	107853	99.00%	1084
LL39	116824	56192	55432	114122	97.69%	2702
LL40	111167	50623	49516	102392	92.11%	8775
LL41	102731	50410	50230	102266	99.55%	465
LL42	91075	43629	43540	89843	98.65%	1232
LL43	102994	48590	48474	102447	99.47%	547
LL44	96963	46123	45886	96239	99.25%	724
LL45	126367	61348	60820	125420	99.25%	947
LL46	110702	51677	49413	107957	97.52%	2745
LL47	132786	58804	56090	124961	94.11%	7825

Sample	Make contigs	Sequences screening	Remove chimeras	Subtracted sequences per sample	% Eliminated sequences per sample	Final sequences per sample
LL49	96005	44924	43820	92679	96.54%	3326
LL50	126199	59053	56285	123427	97.80%	2772
LL51	124359	58561	57727	121811	97.95%	2548
LL52	97974	47890	47815	97483	99.50%	491
LL53	127425	55077	54151	125416	98.42%	2009
LL54	129099	54248	53577	128003	99.15%	1096
LL55	137667	58533	55582	136571	99.20%	1096
LL56	134576	56144	55046	133616	99.29%	960
LL57	113331	33204	33088	109354	96.49%	3977
LL58	153644	61116	59222	150742	98.11%	2902
LL59	84130	39419	39328	83607	99.38%	523
LL60	129967	61607	60839	129058	99.30%	909
LL61	102885	43260	42700	102595	99.72%	290
LL62	109133	46082	45850	108664	99.57%	469
LL63	92869	39006	38561	92365	99.46%	504
LL64	100689	42400	41787	99521	98.84%	1168
LL65	117425	48017	47734	116691	99.37%	734
LL66	109659	46269	44852	109364	99.73%	295
LL67	109439	45306	44150	109075	99.67%	364
LL68	102944	42361	41595	102703	99.77%	241
LL69	108318	40578	39984	96865	89.43%	11453
LL70	131395	62346	62220	130587	99.39%	808
LL71	125820	45710	44361	115765	92.01%	10055
LL72	95233	46728	46621	94598	99.33%	635
LL73	108446	47874	47230	107528	99.15%	918
LL74	132158	55248	54102	129013	97.62%	3145
LL75	130521	55931	55437	130083	99.66%	438
LL76	103480	44358	43948	103013	99.55%	467
LL77	124808	44384	44061	97455	78.08%	27353
LL78	128418	59456	59000	126498	98.50%	1920
LL79	112016	43835	42580	102173	91.21%	9843
LL80	110059	50066	49482	103723	94.24%	6336
LL81	137429	62324	61526	134234	97.68%	3195
LL82	119615	54894	54229	114229	95.50%	5386
LL83	121901	50977	48275	114636	94.04%	7265
LL84	126365	55411	53493	112934	89.37%	13431
LL85	142507	66458	65063	137561	96.53%	4946
LL86	144911	60651	59946	135498	93.50%	9413
LL87	146700	60052	53058	134372	91.60%	12328
LL91	133236	59335	58536	127331	95.57%	5905
LL92	174635	74815	70719	166923	95.58%	7712

Sample	Make contigs	Sequences screening	Remove chimeras	Subtracted sequences per sample	% Eliminated sequences per sample	Final sequences per sample
LL95	122704	57934	57532	119274	97.20%	3430
LL96	193373	80848	73812	178566	92.34%	14807
LL97	159277	68629	64196	146161	91.77%	13116
LL103	115362	48038	47329	114317	99.09%	1045
LL104	138839	48489	44490	125591	90.46%	13248
LL105	157328	64063	58863	150219	95.48%	7109
LL106	160549	67576	61971	159419	99.30%	1130
LL107	155232	66159	60999	153025	98.58%	2207
LL108	130969	55491	51586	129955	99.23%	1014
LL109	102973	40722	40126	102218	99.27%	755
LL110	157253	62665	58787	155826	99.09%	1427
LL111	143281	59964	59024	131561	91.82%	11720
LL112	160232	66692	63267	152851	95.39%	7381
LL113	174925	70474	67321	173764	99.34%	1161
LL114	208346	80518	76033	196310	94.22%	12036
LL117	187027	79427	74004	158458	84.72%	28569
LL119	164167	68374	57082	160852	97.98%	3315
LL120	116875	46313	45103	112824	96.53%	4051
LL121	123914	54852	54077	120075	96.90%	3839
LL122	138638	57788	56407	136678	98.59%	1960
LL123	123728	52289	51161	122906	99.34%	822
LL124	119215	56115	54427	116546	97.76%	2669
LL125	150188	67244	64150	141755	94.39%	8433
LL126	102828	47944	46546	88843	86.40%	13985
LL127	168757	79026	76618	166565	98.70%	2192
LL128	116706	51345	50279	112333	96.25%	4373
LL129	123650	57401	56290	119852	96.93%	3798
LL135	110771	72914	71005	105133	94.91%	5638
LL136	107364	69820	66822	99170	92.37%	8194
LL137	126939	80152	72348	122541	96.54%	4398
LL138	95469	61841	59673	94835	99.34%	634
LL139	97972	67546	65329	96756	98.76%	1216
LL140	105830	72021	69088	103823	98.10%	2007
LL141	114980	76447	70866	113458	98.68%	1522
LL142	112279	62041	61538	111138	98.98%	1141
LL143	112015	73789	65642	108811	97.14%	3204
LL144	149038	91576	69012	145189	97.42%	3849
LL298	112262	56035	55814	73908	65.84%	38354
LL300	156072	94604	93623	105071	67.32%	51001
LL302	114160	68064	67176	73712	64.57%	40448
LL303	140683	88392	84948	96946	68.91%	43737

Sample	Make contigs	Sequences screening	Remove chimeras	Subtracted sequences per sample	% Eliminated sequences per sample	Final sequences per sample
LL304	160778	102356	100795	113412	70.54%	47366
LL306	156954	95802	92463	130764	83.31%	26190
LL307	103065	63913	62573	82001	79.56%	21064
LL308	90914	58799	58532	86449	95.09%	4465
LL309	227262	102826	101070	224784	98.91%	2478
LL310	142384	92945	86189	135603	95.24%	6781
LL311	157776	104575	82654	154609	97.99%	3167
IZ1	110250	76757	74857	98930	89.73%	11320
IZ2	111976	74860	70002	96307	86.01%	15669
IZ3	134582	90485	83159	110333	81.98%	24249
IZ7	140403	90263	82856	109312	77.86%	31091
IZ8	107084	73327	72872	103524	96.68%	3560
IZ9	83847	54840	54204	66510	79.32%	17337
IZ10	107324	72837	71020	94823	88.35%	12501
IZ11	112719	77807	76072	102615	91.04%	10104
IZ12	146786	101581	97687	139542	95.06%	7244
IZ13	110221	74090	69175	97395	88.36%	12826
IZ14	139405	95661	91655	121207	86.95%	18198
IZ15	100071	69473	68797	92492	92.43%	7579
IZ16	118596	81073	77782	103739	87.47%	14857
IZ17	126205	83739	72556	94692	75.03%	31513
IZ18	124440	89125	89063	123370	99.14%	1070
IZ19	92119	65600	65555	91296	99.11%	823
IZ21	122364	84513	81387	111223	90.90%	11141
IZ22	125181	84341	79983	106704	85.24%	18477
IZ23	114783	80089	79644	110072	95.90%	4711
IZ24	106822	70307	65410	86183	80.68%	20639
IZ25	120928	81182	76962	104243	86.20%	16685
IZ26	117160	79848	74454	99486	84.91%	17674
IZ27	130886	89963	88245	118999	90.92%	11887
IZ28	112542	74919	72945	98607	87.62%	13935
IZ29	155214	107821	104272	141209	90.98%	14005
IZ35	193422	101787	93701	132387	68.44%	61035
IZ38	121493	81892	79017	116634	96.00%	4859
IZ39	155433	94221	88341	137047	88.17%	18386
IZ43	124212	86648	85106	121351	97.70%	2861
IZ44	100407	65645	65398	96922	96.53%	3485
IZ45	133424	92537	89081	127916	95.87%	5508
IZ46	94778	67421	65728	90219	95.19%	4559
IZ47	124350	87210	85251	121706	97.87%	2644

Sample	Make contigs	Sequences screening	Remove chimeras	Subtracted sequences per sample	% Eliminated sequences per sample	Final sequences per sample
IZ48	95597	67457	66659	93978	98.31%	1619
IZ51	110091	75712	74231	102699	93.29%	7392
IZ52	115102	81363	80417	110829	96.29%	4273
IZ53	141444	99610	98063	138047	97.60%	3397
IZ54	104851	72949	72478	103218	98.44%	1633
IZ55	111722	76761	76163	107016	95.79%	4706
IZ58	86112	60743	60346	85100	98.82%	1012
IZ59	97571	67190	66898	95823	98.21%	1748
IZ60	91904	63796	63504	89018	96.86%	2886
IZ61	92940	66212	65998	91961	98.95%	979
IZ62	98763	66589	66146	95504	96.70%	3259
IZ68	107487	74088	73957	104840	97.54%	2647
IZ69	117695	81583	81087	115284	97.95%	2411
IZ70	91091	64592	64377	89215	97.94%	1876
IZ71	85754	60723	60627	84848	98.94%	906
IZ72	113754	79959	79428	112266	98.69%	1488
IZ75	93155	65011	63989	89105	95.65%	4050
IZ76	145781	97103	91243	139234	95.51%	6547
IZ77	113497	80726	80566	110983	97.78%	2514
IZ78	135353	92301	90556	125642	92.83%	9711
IZ81	126534	81473	79377	123964	97.97%	2570
IZ82	163341	107822	100896	155143	94.98%	8198
IZ83	151024	104106	102651	148354	98.23%	2670
IZ84	116592	81112	80897	113772	97.58%	2820
IZ85	161108	114801	113952	157650	97.85%	3458
IZ90	147898	90878	90258	113110	76.48%	34788
IZ91	218482	137395	131640	172317	78.87%	46165
IZ92	123859	77369	76698	112921	91.17%	10938
IZ93	121301	75764	74623	93236	76.86%	28065
IZ94	153523	97196	92098	96647	62.95%	56876
IZ103	201637	106958	89778	152081	75.42%	49556
IZ104	161821	94626	92045	100733	62.25%	61088
IZ105	146437	91579	90551	135900	92.80%	10537
IZ106	150921	99471	98178	146414	97.01%	4507
IZ107	141285	88240	87407	136677	96.74%	4608
IZ108	138826	88638	87933	137128	98.78%	1698
IZ119	124659	80944	80367	102292	82.06%	22367
IZ120	194742	112022	109685	126400	64.91%	68342
IZ127	123824	83065	82138	122569	98.99%	1255

Sample	Make contigs	Sequences screening	Remove chimeras	Subtracted sequences per sample	% Eliminated sequences per sample	Final sequences per sample
IZ128	124246	80923	79943	123080	99.06%	1166
IZ129	117444	80213	79591	116342	99.06%	1102
IZ130	113198	80957	80722	111518	98.52%	1680
IZ134	116161	77404	75690	109009	93.84%	7152
IZ135	116999	83462	83131	116104	99.24%	895
IZ141	223486	93272	89773	207355	92.78%	16131
IZ142	99015	60087	59086	94091	95.03%	4924
IZ149	139179	85486	83345	137802	99.01%	1377
IZ312	165838	105529	93038	146790	88.51%	19048
IZ313	96835	64833	63578	87265	90.12%	9570
IZ314	120751	46183	46001	109312	90.53%	11439
IZ315	100752	69256	69006	98067	97.34%	2685
IZ317	76135	53203	53044	74787	98.23%	1348
IZ318	174108	114901	106698	153496	88.16%	20612
IZ319	231691	144586	133649	211418	91.25%	20273
IZ320	164273	103486	96013	152959	93.11%	11314
IZ321	182166	119357	111797	176455	96.86%	5711
IZ322	398837	115750	112847	393585	98.68%	5252
IZ323	170657	114220	109268	163200	95.63%	7457
IZ324	187101	81376	80970	184946	98.85%	2155
IZ325	175976	117941	114072	160512	91.21%	15464
IZ326	144928	89999	86917	74750	51.58%	70178
IZ328	98141	59968	58724	74458	75.87%	23683
IZ329	258189	98022	93295	230225	89.17%	27964
IZ330	139242	87341	85570	130908	94.01%	8334
IZ331	161445	103440	99667	102118	63.25%	59327
IZ335	176551	112927	100811	174973	99.11%	1578
IZ336	131034	88416	87770	126404	96.47%	4630
IZ337	146270	92713	90285	143950	98.41%	2320
IZ338	122886	74103	73435	116090	94.47%	6796
IZ339	146075	87926	87207	135050	92.45%	11025
IZ340	153234	93441	81310	152017	99.21%	1217

Table S2. Izaña sampling location. Bacterial phyla and genera number of sequences and relative abundances by season. Only genera present at both sampling sites have been included.

Phylum	Genus	Season: Spring Number of Sequences	Spring % Relative Abundance	Summer Number of Sequences	Summer % Relative Abundance	Fall Number of Sequences	Fall % Relative Abundance	Winter Number of Sequences	Winter % Relative Abundance
Actinobacteria	*Citricoccus*	0	0.00%	17	0.00%	327	0.23%	45	0.01%
	Corynebacterium	1,778	0.49%	396	0.08%	1,554	1.11%	147	0.05%
	Kocuria	239	0.07%	45	0.01%	573	0.41%	35	0.01%
	Microbacteriaceae (unclassified genus)	525	0.15%	12,824	2.64%	451	0.32%	975	0.32%
	Microbacterium	976	0.27%	11,019	2.27%	1,443	1.03%	7,656	2.50%
	Nocardioides	2,246	0.62%	662	0.14%	190	0.14%	61	0.02%
	Other	7,314	2.03%	3,866	0.80%	6,994	4.98%	3,616	1.18%
Bacteroidetes	*Algoriphagus*	1,993	0.55%	9,346	1.93%	1,186	0.84%	11,063	3.61%
	Bacteroidetes (unclassified class)	6,496	1.80%	1,069	0.22%	582	0.41%	2,797	0.91%
	Chitinophagaceae (unclassified genus)	13,453	3.73%	29,003	5.98%	370	0.26%	546	0.18%
	Chryseobacterium	369	0.10%	68	0.01%	476	0.34%	593	0.19%
	Cloacibacterium	12,591	3.50%	1,966	0.41%	52	0.04%	86	0.03%
	Flavobacteriaceae (unclassified genus)	380	0.11%	84	0.02%	105	0.07%	194	0.06%
	Flavobacterium	572	0.16%	14,782	3.05%	407	0.29%	3,072	1.00%
	Hymenobacter	47	0.01%	19	0.00%	16	0.01%	0	0.00%
	Pedobacter	5,884	1.63%	1,615	0.33%	12	0.01%	1,950	0.64%
	Sediminibacterium	101	0.03%	3,757	0.77%	14,412	10.26%	38,025	12.41%
	Sphingobacteriales (unclassified family)	297	0.08%	10	0.00%	23	0.02%	82	0.03%
	Other	7,324	2.03%	6,544	1.35%	4,941	3.52%	4,741	1.55%

Phylum	Genus	Season							
		Spring		Summer		Fall		Winter	
		Number of Sequences	% Relative Abundance	Number of Sequences	% Relative Abundance	Number of Sequences	% Relative Abundance	Number of Sequences	% Relative Abundance
Chlamydiae	*Parachlamydiaceae (unclassified genus)*	82	0.02%	193	0.04%	309	0.22%	3,804	1.24%
	Other	0	0.00%	12	0.00%	145	0.10%	1,093	0.36%
Firmicutes	*Alicyclobacillus*	6,753	1.87%	1	0.00%	2	0.00%	0	0.00%
	Bacillus	4,733	1.31%	422	0.09%	647	0.46%	1,149	0.37%
	Enterococcus	13,878	3.85%	157	0.03%	493	0.35%	18	0.01%
	Lachnospiraceae (unclassified genus)	10,794	3.00%	34	0.01%	275	0.20%	5	0.00%
	Other	10,836	3.01%	10,192	2.10%	7,450	5.30%	2,669	0.87%
Planctomycetes	*Planctomycetaceae (unclassified genus)*	49	0.01%	301	0.06%	1,847	1.31%	1,063	0.35%
	Other	2	0.00%	146	0.03%	398	0.28%	363	0.12%
Proteobacteria	*Achromobacter*	8,340	2.32%	707	0.15%	163	0.12%	2,348	0.77%
	Acidovorax	595	0.17%	22,580	4.65%	333	0.24%	193	0.06%
	Acinetobacter	175	0.05%	129	0.03%	449	0.32%	1,886	0.62%
	Alishewanella	8,854	2.46%	340	0.07%	763	0.54%	1,987	0.65%
	Amaricoccus	681	0.19%	524	0.11%	367	0.26%	787	0.26%
	Bdellovibrio	3,462	0.96%	9	0.00%	39	0.03%	3,335	1.09%
	Blastomonas	162	0.04%	94,551	19.49%	8,441	6.01%	5,357	1.75%
	Brevundimonas	11,751	3.26%	14,072	2.90%	2,055	1.46%	7,145	2.33%
	Brucella	8,370	2.32%	2,206	0.45%	455	0.32%	3,436	1.12%
	Caulobacteraceae (unclassified genus)	54	0.01%	287	0.06%	610	0.43%	73	0.02%
	Cellvibrio	113,266	31.44%	3,071	0.63%	73	0.05%	94,155	30.73%
	Delftia	1,008	0.28%	379	0.08%	671	0.48%	56	0.02%
	Gammaproteobacteria (unclassified genus)	237	0.07%	437	0.09%	666	0.47%	957	0.31%
	Halomonas	0	0.00%	588	0.12%	312	0.22%	4	0.00%
	Hydrogenophaga	311	0.09%	10,943	2.26%	719	0.51%	816	0.27%

Phylum	Genus	Season							
		Spring		Summer		Fall		Winter	
		Number of Sequences	% Relative Abundance	Number of Sequences	% Relative Abundance	Number of Sequences	% Relative Abundance	Number of Sequences	% Relative Abundance
	Limnobacter	14	0.00%	145,930	30.08%	5,545	3.95%	43	0.01%
	Mesorhizobium	290	0.08%	4,334	0.89%	1,140	0.81%	4,870	1.59%
	Methylobacterium	1,201	0.33%	1,708	0.35%	3,861	2.75%	1,758	0.57%
	Methyloversatilis	187	0.05%	3,745	0.77%	190	0.14%	509	0.17%
	Nevskia	324	0.09%	284	0.06%	652	0.46%	609	0.20%
	Paracoccus	2,771	0.77%	993	0.20%	3,891	2.77%	6,819	2.23%
	Parvibaculum	6,589	1.83%	187	0.04%	31	0.02%	699	0.23%
	Phenylobacterium	1,986	0.55%	288	0.06%	688	0.49%	2,905	0.95%
	Pseudomonadaceae (unclassified genus)	3,824	1.06%	10,379	2.14%	9,703	6.91%	5,381	1.76%
	Pseudomonas	224	0.06%	864	0.18%	197	0.14%	587	0.19%
	Pseudoxanthomonas	16,937	4.70%	15,389	3.17%	4,338	3.09%	6,783	2.21%
	Reyranella (unclassified genus)	5,670	1.57%	565	0.12%	5,760	4.10%	14,316	4.67%
	Rhizobiaceae (unclassified genus)	587	0.16%	881	0.18%	152	0.11%	565	0.18%
	Rhizobium	6,902	1.92%	1,046	0.22%	293	0.21%	9,680	3.16%
	Shewanella	43	0.01%	6,928	1.43%	51	0.04%	82	0.03%
	Sphingobium	1,924	0.53%	800	0.16%	291	0.21%	479	0.16%
	Sphingomonas	4,452	1.24%	3,788	0.78%	24,101	17.15%	9,196	3.00%
	Sphingopyxis	1,991	0.55%	2,378	0.49%	1,685	1.20%	749	0.24%
	Stenotrophomonas	4,944	1.37%	1,876	0.39%	130	0.09%	1,342	0.44%
	Thiobacillus	115	0.03%	0	0.00%	0	0.00%	286	0.09%
	Xanthobacter	465	0.13%	214	0.04%	80	0.06%	914	0.30%
	Other	27,255	7.57%	21,566	4.45%	14,341	10.21%	27,381	8.94%
Bacteria_ unclassified	*Unclassified*	4,569	1.27%	1,571	0.32%	1,597	1.14%	2,079	0.68%

Table S3. Laguna sampling location. Bacterial phyla and genera number of sequences and relative abundances by season. Only genera present at both sampling sites have been included.

		Season							
		Spring		Summer		Fall		Winter	
**Phylum**	**Genus**	**Number of Sequences**	**% Relative Abundance**	**Number of Sequences**	**% Relative Abundance**	**Number of Sequences**	**% Relative Abundance**	**Number of Sequences**	**% Relative Abundance**
Actinobacteria	*Citricoccus*	0	0.00%	6,072	1.94%	50	0.03%	16	0.01%
	Corynebacterium	596	0.33%	1,064	0.34%	703	0.42%	83	0.04%
	Kocuria	68	0.04%	227	0.07%	293	0.17%	10,459	5.52%
	Microbacteriaceae (unclassified genus)	358	0.20%	293	0.09%	344	0.20%	226	0.12%
	Microbacterium	920	0.51%	983	0.31%	592	0.35%	699	0.37%
	Nocardioides	904	0.50%	2,168	0.69%	4,748	2.80%	883	0.47%
	Other	2,753	1.53%	5,555	1.77%	3,646	2.15%	3,773	1.99%
Bacteroidetes	*Algoriphagus*	751	0.42%	49	0.02%	0	0.00%	1,110	0.59%
	Bacteroidetes (unclassified class)	3,428	1.91%	10,251	3.27%	1,389	0.82%	669	0.35%
	Chitinophagaceae (unclassified genus)	4,602	2.56%	9,550	3.05%	1,278	0.75%	917	0.48%
	Chryseobacterium	3,431	1.91%	10	0.00%	128	0.08%	401	0.21%
	Cloacibacterium	220	0.12%	150	0.05%	203	0.12%	706	0.37%
	Flavobacteriaceae (unclassified genus)	146	0.08%	3,707	1.18%	208	0.12%	543	0.29%
	Flavobacterium	1,605	0.89%	771	0.25%	171	0.10%	1,975	1.04%
	Hymenobacter	5	0.00%	99	0.03%	1,727	1.02%	1	0.00%
	Pedobacter	1,582	0.88%	1	0.00%	162	0.10%	1,910	1.01%
	Sediminibacterium	4,144	2.31%	923	0.29%	437	0.26%	10,425	5.50%
	Sphingobacteriales (unclassified family)	132	0.07%	3,540	1.13%	68	0.04%	106	0.06%
	Other	3,883	2.16%	2,977	0.95%	2,088	1.23%	2,992	1.58%

Phylum	Genus	Season							
		Spring		Summer		Fall		Winter	
		Number of Sequences	% Relative Abundance	Number of Sequences	% Relative Abundance	Number of Sequences	% Relative Abundance	Number of Sequences	% Relative Abundance
Chlamydiae	*Parachlamydiaceae (unclassified genus)*	288	0.16%	263	0.08%	18	0.01%	792	0.42%
	Other	105	0.06%	1	0.00%	0	0.00%	162	0.09%
Firmicutes	*Alicyclobacillus*	0	0.00%	1	0.00%	0	0.00%	0	0.00%
	Bacillus	2,141	1.19%	5,039	1.61%	241	0.14%	867	0.46%
	Enterococcus	67	0.04%	4	0.00%	9	0.01%	55	0.03%
	Lachnospiraceae (unclassified genus)	21	0.01%	116	0.04%	16	0.01%	42	0.02%
	Other	1,726	0.96%	10,100	3.22%	5,367	3.17%	1,383	0.73%
Planctomycetes	*Planctomycetaceae (unclassified genus)*	191	0.11%	261	0.08%	1,406	0.83%	241	0.13%
	Other	42	0.02%	41	0.01%	36	0.02%	24	0.01%
Proteobacteria	*Achromobacter*	1,835	1.02%	555	0.18%	441	0.26%	1,807	0.95%
	Acidovorax	72	0.04%	1,756	0.56%	1,328	0.78%	784	0.41%
	Acinetobacter	442	0.25%	24,224	7.73%	14,216	8.39%	3,260	1.72%
	Alishewanella	1,031	0.57%	6,357	2.03%	3,165	1.87%	27	0.01%
	Amaricoccus	1,863	1.04%	150	0.05%	6	0.00%	8,946	4.72%
	Bdellovibrio	1,514	0.84%	781	0.25%	1,667	0.98%	703	0.37%
	Blastomonas	125	0.07%	99	0.03%	1,185	0.70%	112	0.06%
	Brevundimonas	2,701	1.50%	19,101	6.10%	7,813	4.61%	2,108	1.11%
	Brucella	5,199	2.90%	10,182	3.25%	9,452	5.58%	4,057	2.14%
	Caulobacteraceae (unclassified genus)	416	0.23%	24,569	7.84%	8,687	5.13%	1,162	0.61%
	Cellvibrio	50,463	28.11%	737	0.24%	212	0.13%	37,240	19.66%
	Delftia	952	0.53%	1,728	0.55%	437	0.26%	2,376	1.25%
	Gammaproteobacteria (unclassified genus)	235	0.13%	3,424	1.09%	863	0.51%	1,327	0.70%
	Halomonas	18	0.01%	3,184	1.02%	462	0.27%	55	0.03%
	Hydrogenophaga	880	0.49%	665	0.21%	408	0.24%	3,619	1.91%

Phylum	Genus	Season							
		Spring		Summer		Fall		Winter	
		Number of Sequences	% Relative Abundance	Number of Sequences	% Relative Abundance	Number of Sequences	% Relative Abundance	Number of Sequences	% Relative Abundance
	Limnobacter	3	0.00%	2,677	0.85%	30	0.02%	1	0.00%
	Mesorhizobium	1,561	0.87%	124	0.04%	19,239	11.36%	7,850	4.14%
	Methylobacterium	2,961	1.65%	16,041	5.12%	4,006	2.37%	6,795	3.59%
	Methyloversatilis	156	0.09%	3,702	1.18%	1,421	0.84%	204	0.11%
	Nevskia	178	0.10%	6,700	2.14%	792	0.47%	930	0.49%
	Paracoccus	2,335	1.30%	15,430	4.93%	11,197	6.61%	5,493	2.90%
	Parvibaculum	2,091	1.16%	610	0.19%	17	0.01%	246	0.13%
	Phenylobacterium	1,809	1.01%	1,543	0.49%	955	0.56%	2,132	1.13%
	Pseudomonadaceae (unclassified genus)	5,536	3.08%	3,397	1.08%	6,867	4.05%	4,876	2.57%
	Pseudomonas	14,783	8.24%	1,382	0.44%	162	0.10%	494	0.26%
	Pseudoxanthomonas	10,531	5.87%	27,244	8.70%	13,934	8.23%	10,883	5.74%
	Reyranella (unclassified genus)	3,635	2.03%	2,451	0.78%	1,005	0.59%	2,167	1.14%
	Rhizobiaceae (unclassified genus)	796	0.44%	4,223	1.35%	307	0.18%	1,972	1.04%
	Rhizobium	5,422	3.02%	2,495	0.80%	729	0.43%	1,593	0.84%
	Shewanella	21	0.01%	0	0.00%	37	0.02%	0	0.00%
	Sphingobium	954	0.53%	4,023	1.28%	3,077	1.82%	2,478	1.31%
	Sphingomonas	2,384	1.33%	4,913	1.57%	4,003	2.36%	3,252	1.72%
	Sphingopyxis	1,934	1.08%	11,677	3.73%	3,678	2.17%	4,312	2.28%
	Stenotrophomonas	2,117	1.18%	339	0.11%	231	0.14%	339	0.18%
	Thiobacillus	434	0.24%	18	0.01%	0	0.00%	2,931	1.55%
	Xanthobacter	700	0.39%	5,588	1.78%	3,644	2.15%	341	0.18%
	Other	15,359	8.56%	34,979	11.17%	16,802	9.92%	18,586	9.81%
Bacteria_unclassified	*Unclassified*	1,951	1.09%	1,952	0.62%	1,564	0.92%	2,538	1.34%

Table S4. Bacterial phyla (and Proteobacteria classes) number of sequences (Nº Seq.) and relative abundance by sampling location and season. Supporting data for **Figure 3.A**.

		Sampling location			
		Izaña		La Laguna	
**Season**	**Phylum**	**Nº Seq.**	**% Rel. Abundance**	**Nº Seq.**	**% Rel. Abundance**
Spring	Actinobacteria	13,078	3.62%	5,599	3.08%
	Bacteroidetes	49,507	13.71%	23,929	13.15%
	Firmicutes	46,994	13.01%	3,955	2.17%
	Alphaproteobacteria	70,179	19.43%	46,104	25.34%
	Betaproteobacteria	18,550	5.14%	7,267	3.99%
	Gammaproteobacteria	152,867	42.33%	88,228	48.50%
	Deltaproteobacteria	3,546	0.98%	1,585	0.87%
	Other identified bacteria	1,879	0.52%	3,312	1.82%
	Unclassified bacteria	4,569	1.27%	1,951	1.07%
Summer	Actinobacteria	28,829	5.91%	16,362	5.17%
	Bacteroidetes	68,263	14.00%	32,028	10.11%
	Firmicutes	10,806	2.22%	15,260	4.82%
	Alphaproteobacteria	134,724	27.63%	137,631	43.45%
	Betaproteobacteria	192,821	39.54%	19,663	6.21%
	Gammaproteobacteria	46,660	9.57%	87,549	27.64%
	Deltaproteobacteria	141	0.03%	1,921	0.61%
	Other identified bacteria	3,802	0.78%	4,372	1.38%
	Unclassified bacteria	1,571	0.32%	1,952	0.62%
Fall	Actinobacteria	11,532	8.04%	10,376	6.02%
	Bacteroidetes	22,582	15.75%	7,859	4.56%
	Firmicutes	8,867	6.18%	5,633	3.27%
	Alphaproteobacteria	60,056	41.88%	88,755	51.46%
	Betaproteobacteria	11,500	8.02%	8,180	4.74%
	Gammaproteobacteria	21,005	14.65%	43,064	24.97%
	Deltaproteobacteria	208	0.15%	2,061	1.20%
	Other identified bacteria	6,069	4.23%	4,966	2.88%
	Unclassified bacteria	1,597	1.11%	1,564	0.91%
Winter	Actinobacteria	12,535	3.96%	16,139	8.35%
	Bacteroidetes	63,149	19.97%	21,755	11.25%
	Firmicutes	3,841	1.21%	2,347	1.21%
	Alphaproteobacteria	83,952	26.55%	63,282	32.74%
	Betaproteobacteria	6,969	2.20%	15,732	8.14%
	Gammaproteobacteria	123,868	39.18%	65,034	33.64%
	Deltaproteobacteria	3,376	1.07%	823	0.43%
	Other identified bacteria	16,410	5.19%	5,658	2.93%
	Unclassified bacteria	2,079	0.66%	2,538	1.31%

Table S5. Bacterial phyla (and Proteobacteria classes) number of sequences (Nº Seq) and relative abundance (**%RA**) relative to season and wind back trajectories. Supporting data for **Figure 3.B**.

		**Wind backward trajectory**							
		**African**		**European**		**Marine**		**Tropical**	
**Season**	**Phylum**	**NºSeq.**	**%RA**	**NºSeq.**	**%RA**	**NºSeq.**	**%RA**	**NºSeq.**	**%RA**
Spring	Actinobacteria	2,084	2.69%	1,464	4.17%	12,865	3.38%	2,264	4.57%
	Bacteroidetes	5,716	7.37%	6,728	19.16%	54,753	14.38%	6,239	12.59%
	Firmicutes	3,466	4.47%	416	1.18%	9,900	2.60%	37,167	75.00%
	Alphaproteobacteria	10,878	14.02%	6,098	17.37%	99,281	26.07%	26	0.05%
	Betaproteobacteria	3,273	4.22%	1,245	3.55%	17,535	4.60%	3,764	7.60%
	Gammaproteobacteria	50,076	64.54%	17,201	48.98%	173,735	45.62%	83	0.17%
	Deltaproteobacteria	1,862	2.40%	254	0.72%	3,015	0.79%	0	0.00%
	Other ID bacteria	147	0.19%	1,352	3.85%	3,692	0.97%	0	0.00%
	Unclassified bacteria	92	0.12%	358	1.02%	6,057	1.59%	13	0.03%
Summer	Actinobacteria	10,093	9.42%	2,254	2.71%	28,033	5.34%	4,811	5.38%
	Bacteroidetes	8,838	8.25%	8,597	10.34%	80,525	15.35%	2,331	2.61%
	Firmicutes	758	0.71%	8,152	9.80%	15,635	2.98%	1,521	1.70%
	Alphaproteobacteria	49,723	46.40%	38,566	46.37%	165,255	31.50%	18,811	21.02%
	Betaproteobacteria	28,143	26.26%	4,818	5.79%	127,027	24.22%	52,496	58.67%
	Gammaproteobacteria	8,814	8.23%	18,179	21.86%	97,793	18.64%	9,423	10.53%
	Deltaproteobacteria	92	0.09%	186	0.22%	1,778	0.34%	6	0.01%
	Other ID bacteria	611	0.57%	1,512	1.82%	6,009	1.15%	42	0.05%
	Unclassified bacteria	82	0.08%	912	1.10%	2,497	0.48%	32	0.04%
Fall	Actinobacteria	8,956	10.08%	6,632	8.79%	4,363	3.69%	1,957	5.86%
	Bacteroidetes	8,726	9.82%	9,339	12.38%	7,104	6.01%	5,272	15.78%
	Firmicutes	6,562	7.39%	2,867	3.80%	3,798	3.21%	1,273	3.81%
	Alphaproteobacteria	30,483	34.31%	38,765	51.37%	63,362	53.63%	16,201	48.48%
	Betaproteobacteria	6,486	7.30%	2,784	3.69%	9,342	7.91%	1,068	3.20%
	Gammaproteobacteria	21,595	24.31%	12,038	15.95%	23,774	20.12%	6,662	19.94%
	Deltaproteobacteria	274	0.31%	79	0.10%	1,895	1.60%	21	0.06%
	Other ID bacteria	4,606	5.18%	2,537	3.36%	3,078	2.61%	814	2.44%
	Unclassified bacteria	1,155	1.30%	416	0.55%	1,441	1.22%	149	0.45%
Winter	Actinobacteria	16,549	7.32%	2,868	2.74%	9,224	5.21%	33	2.40%
	Bacteroidetes	37,367	16.52%	19,352	18.48%	27,953	15.78%	232	16.87%
	Firmicutes	3,832	1.69%	241	0.23%	2,092	1.18%	23	1.67%
	Alphaproteobacteria	67,307	29.75%	32,786	31.30%	46,888	26.47%	253	18.40%
	Betaproteobacteria	8,753	3.87%	3,938	3.76%	9,969	5.63%	41	2.98%
	Gammaproteobacteria	79,527	35.16%	38,030	36.31%	70,715	39.92%	630	45.82%
	Deltaproteobacteria	331	0.15%	99	0.09%	3,755	2.12%	14	1.02%
	Other ID bacteria	10,335	4.57%	6,289	6.00%	5,343	3.02%	101	7.35%
	Unclassified bacteria	2,216	0.98%	1,138	1.09%	1,215	0.69%	48	3.49%

Table S6. Dunn's Z test for the comparison of the observed microbial richness between seasons in La Laguna samples. Supporting data for **Figure S1.**

Comparison	Dunn's Z test	Adj. p value
Spring - Summer	-1.1693	0.72686
Spring - Fall	4.1497	0.00010
Spring - Winter	2.4934	0.03796
Summer - Fall	4.9458	0.00000
Summer - Winter	3.4320	0.00180
Fall - Winter	-1.5622	0.35471

Table S7. Dunn's Z test for the comparison of the observed microbial richness between different wind back trajectories in La Laguna. Supporting data for **Figure S1**

Comparison	Dunn's Z test	Adj. p value
African - European	-0.7372	1.00000
African - Marine	-2.9128	0.01074
African - Tropical	1.2852	0.59618
European - Marine	-2.1834	0.08703
European - Tropical	1.6730	0.28299
Marine - Tropical	2.6311	0.02554

Table S8. Dunn's Z test for the comparison of the observed microbial richness between different wind back trajectories in Izaña. Supporting data for **Figure S1**

Comparison	Dunn's Z test	Adj. p value
African - European	0.2246	1.00000
African - Marine	1.7422	0.24443
African - Tropical	2.8945	0.01139
European - Marine	0.7875	1.00000
European - Tropical	1.7835	0.22354
Marine - Tropical	1.7373	0.24699

Table S9. Number of sequences (Nº Seq.) and Relative Abundance (%RA); considering seasons and wind back trajectories for the most frequent genera in each category. These data are represented in **Figure 5**.

		**Season**							
		**Spring**		**Summer**		**Fall**		**Winter**	
**Wind back trajectory**	**Genus**	**Nº Seq.**	**% RA**	**Nº Seq.**	**% RA**	**Nº Seq.**	**% RA**	**Nº Seq.**	**% RA**
African	*Acinetobacter*	26	0.03%	167	0.16%	8,944	10.18%	2,615	1.17%
	Blastomonas	1	0.00%	29,970	28.10%	2,850	3.24%	3,616	1.61%
	Cellvibrio	43,348	55.91%	599	0.56%	108	0.12%	51,838	23.11%
	Limnobacter	0	0.00%	22,601	21.19%	2,952	3.36%	11	0.00%
European	*Cellvibrio*	1,450	4.14%	181	0.22%	38	0.05%	30,404	29.27%
	Pseudomonas	12,312	35.14%	50	0.06%	17	0.02%	120	0.12%
	Pseudoxanthomonas	825	2.35%	8,828	10.62%	2,146	2.85%	2,584	2.49%
	Sediminibacterium	265	0.76%	672	0.81%	5,958	7.93%	13,620	13.11%
Marine	*Blastomonas*	259	0.07%	57,730	11.05%	2,371	2.02%	751	0.43%
	Cellvibrio	118,930	31.30%	704	0.13%	116	0.10%	49,131	27.97%
	Limnobacter	16	0.00%	83,986	16.08%	2,391	2.04%	11	0.01%
	Mesorhizobium	1,682	0.44%	794	0.15%	13,553	11.57%	4,164	2.37%
Tropical	*Alicyclobacillus*	6,753	13.63%	0	0.00%	1	0.00%	0	0.00%
	Cloacibacterium	6,239	12.59%	12	0.01%	0	0.00%	3	0.22%
	Enterococcus	13,793	27.83%	0	0.00%	49	0.15%	1	0.07%
	Lachnospiraceae	10,746	21.68%	0	0.00%	125	0.38%	0	0.00%
	Limnobacter	1	0.00%	41,999	46.95%	195	0.59%	1	0.07%
	Pseudomonadaceae	59	0.12%	1,293	1.45%	3,670	11.06%	352	26.15%
	Sphingomonas	0	0.00%	28	0.03%	8,470	25.52%	34	2.53%

Figure S1. Alpha diversity measures (Observed richness, Chao 1, and Shannon indexes) at both locations relative to season (**A**) and wind back trajectories (**B**). OTUs that did not appear more than 5 times in more than half of the samples were removed. Five most predominant phyla were selected for the analysis.

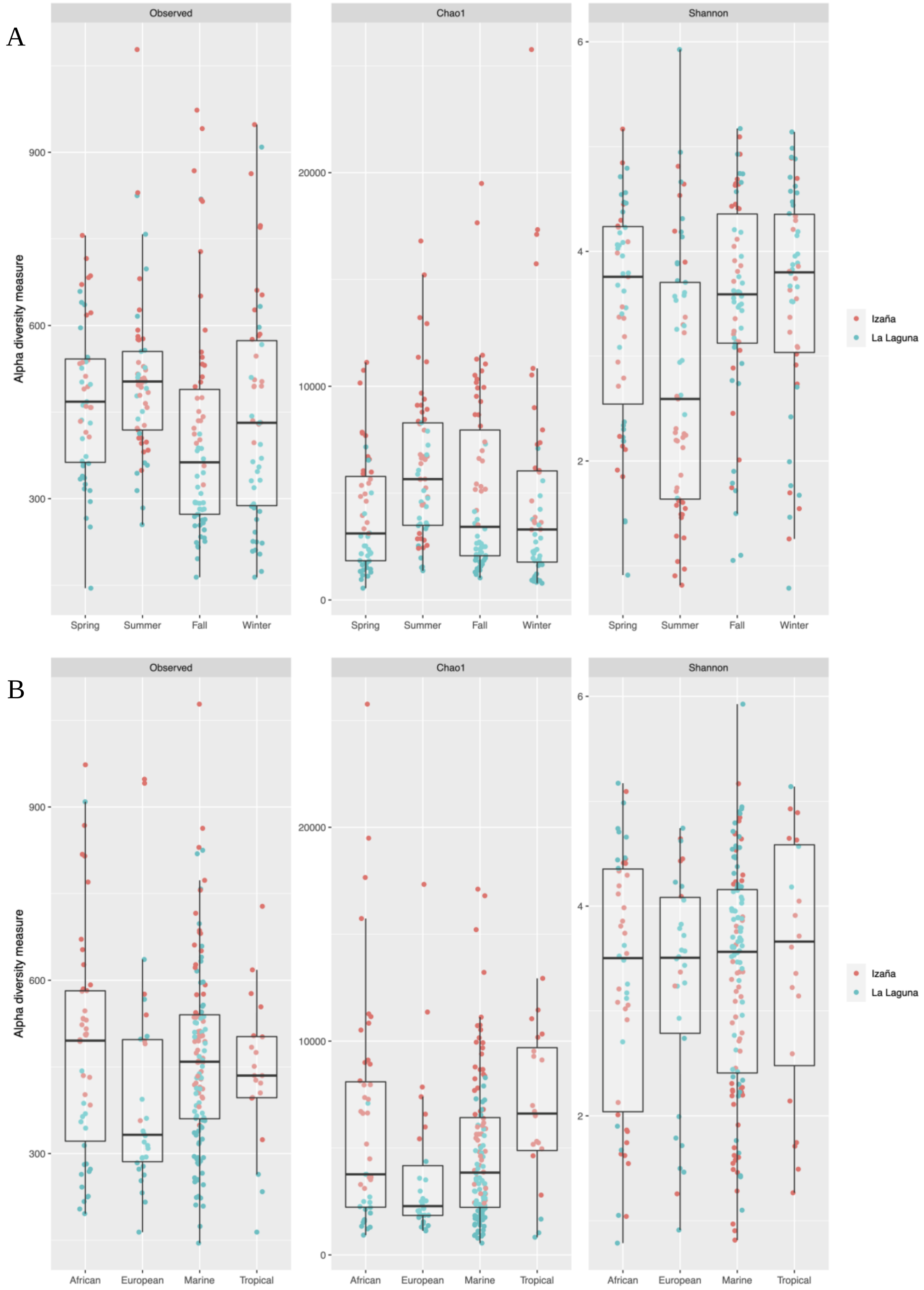

Figure S2. Analysis of similarity (ANOSIM) of bacterial communities on a phylum level based on Bray-Curtis algorithm. Samples were grouped based on the sampling location and season. (R ANOSIM = 0.0.0519-0.546, all p-values < 0.01, except for Spring (0.0701)).

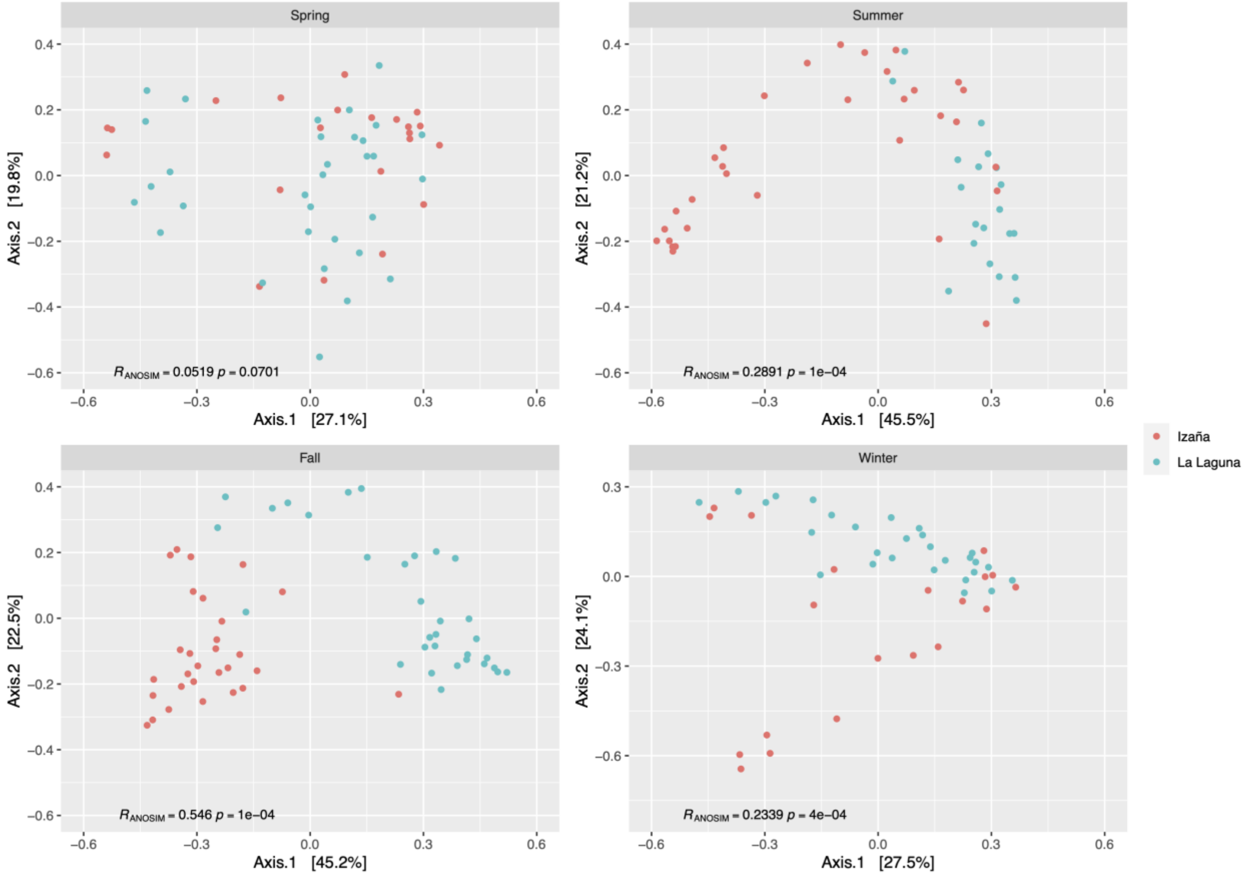

Figure S3. Principal coordinate analysis of species according to their predominance across seasons. OTUs that did not appear more than 5 times in more than half of the samples were removed. Five most predominant phyla were selected for the analysis.

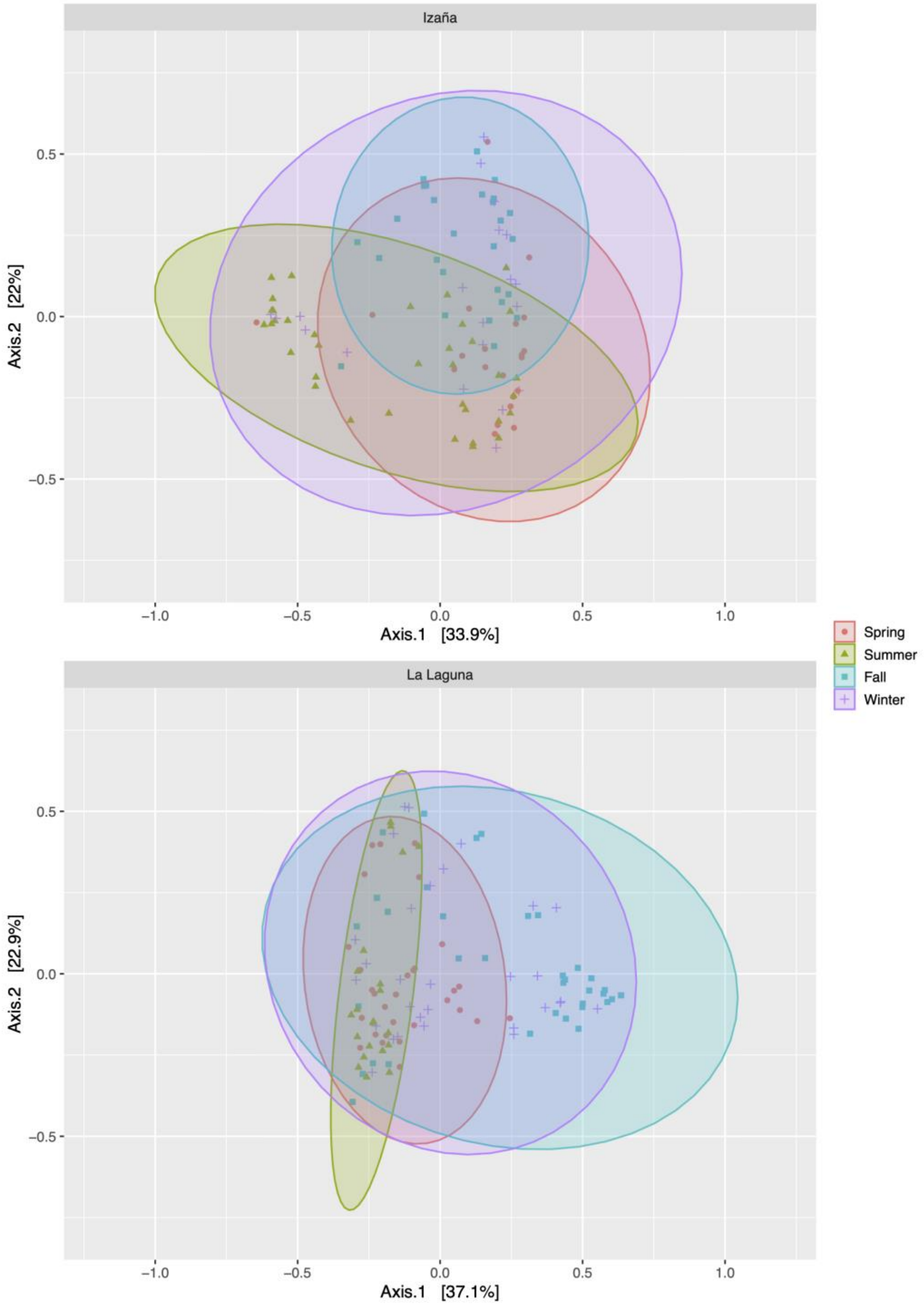

Supplement: Supplementary file 1 [file Data_Sheet_1.PDF]
